# Síndrome do Bloqueio de Ramo Esquerdo Doloroso em Paciente Encaminhada para Estudo Eletrofisiológico: Um Relato de Caso

**DOI:** 10.36660/abc.20190295

**Published:** 2020-05-11

**Authors:** José Nunes de Alencar, Marcel Henrique Sakai, Saulo Rodrigo Ramalho de Moraes, Elano Sousa da Frota, Claudio Cirenza, Angelo Amato Vincenzo de Paola

**Affiliations:** 1 Universidade Federal de São Paulo Escola Paulista de Medicina São PauloSP Brasil Universidade Federal de São Paulo Escola Paulista de Medicina, São Paulo, SP - Brasil

**Keywords:** Bloqueio Cardíaco, Dor no Peito, Doença Arterial Coronariana/fisiopatologia, Eletrofisiologia Cardíaca, Eletrocardiografia, Ecocardiografia

## Introdução

O desenvolvimento de dor precordial associada ao bloqueio de ramo esquerdo (BRE) intermitente na ausência de doença arterial coronariana tem sido descrito na literatura como síndrome do bloqueio de ramo esquerdo doloroso. O mecanismo responsável pela dor precordial é desconhecido, mas a principal hipótese atualmente está relacionada à dissincronia cardíaca aguda.

Nessa síndrome, o BRE ocorre quando a duração do ciclo é igual ou inferior ao período refratário do ramo esquerdo, principalmente durante o esforço físico. A dor torácica na síndrome do BRE doloroso pode variar entre um leve desconforto a uma condição incapacitante.

Esse relato descreve uma paciente com BRE frequência-dependente típico associado com dor torácica, encaminhada ao estudo eletrofisiológico (EEF) sem evidências de arritmias.

## Relato de caso

Paciente do sexo feminino, 41 anos, com histórico de hipertensão controlada e dois anos de palpitações associadas com dor torácica desencadeada por esforço mínimo durante atividades cotidianas, que persistia por até 2 horas. A dor torácica foi descrita como uma sensação de pressão, que irradiava para o braço esquerdo, associada a náusea e dispneia. Os episódios foram caracterizados por início súbito, sem pródromos, com melhora espontânea. Inicialmente, a paciente foi tratada com atenolol 25 mg (duas vezes ao dia), com alívio parcial dos sintomas. Não havia histórico familiar de síncope inexplicada ou morte cardíaca súbita. O exame físico foi normal. O ECG de 12 derivações durante a crise revelou taquicardia de complexo alargado com bloqueio de ramo esquerdo completo (BRE), eixo inferior e onda P compatível com ritmo sinusal. Mesmo assim, a paciente foi referia ao EEF, que não mostrou substratos arritmogênicos.

**Figura 1:**
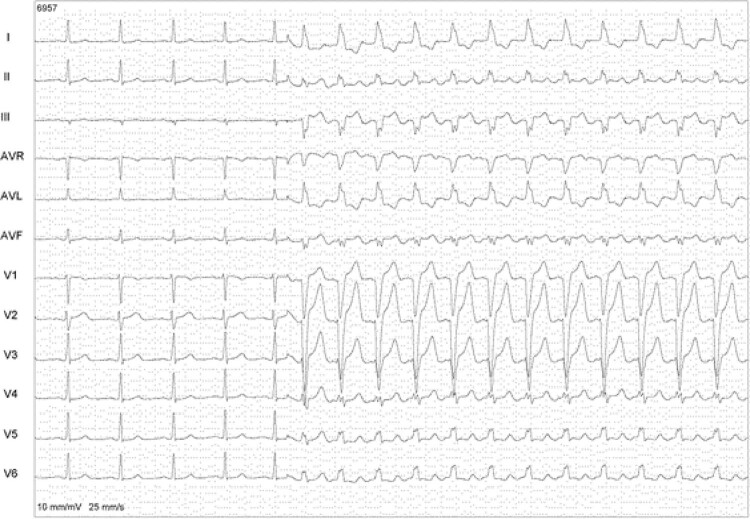
- ECG de 12 derivações demonstrando o início da dor imediatamente após o bloqueio de ramo esquerdo.

Entretanto, no início da estimulação atrial contínua a 600 ms, foi observado um BRE frequência dependente. Imediatamente após o BRE, a paciente, que não havia sido mantida sedada, começou a se queixar dos mesmos sintomas já descritos. O BRE persistiu por alguns minutos e desapareceu espontaneamente, concomitantemente com o alívio da dor. O ECG é apresentado na Figura 1 . Trata-se de um BRE de 3º grau típico, com duração do complexo QRS de 138 ms, eixo superior e onda P sinusal.

**Figura 2:**
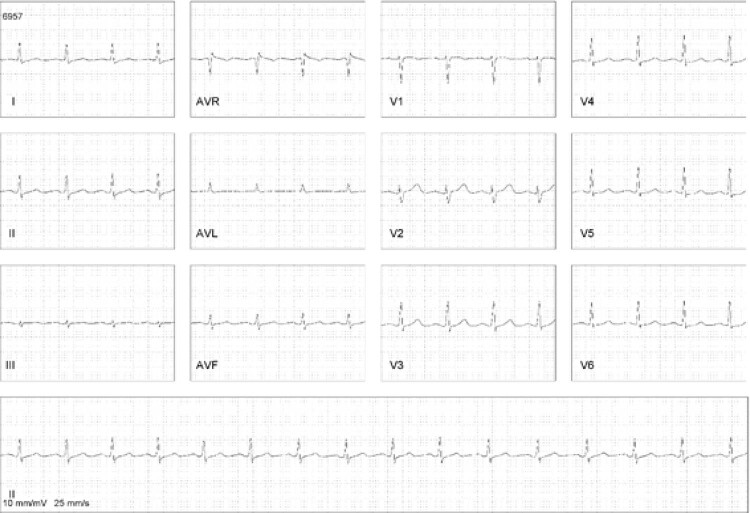
- ECG basal de 12 derivações normal.

O ECG basal de 12 derivações estava normal ( Figura 2 ). O monitoramento por Holter de 24 horas revelou que a FC basal variou entre 56 e 116 bpm durante as atividades cotidianas, sem evidência de BRE. Tanto a ecocardiografia transtorácica quanto a ressonância magnética cardíaca mostraram função sistólica normal sem doença miocárdica ou valvar. Todas as câmaras cardíacas estavam com tamanho normal. Um teste de esforço revelou o desenvolvimento de bloqueio de ramo esquerdo associado com a dor torácica. A angiotomografia descartou doença arterial coronariana e defeitos de perfusão do miocárdio com diripidamol. Atualmente, a paciente está recebendo atenolol 50 mg (duas vezes ao dia) e não houve recorrências de palpitações ou dor precordial no acompanhamento de seis meses.

## Discussão

Em 1946, foi publicado o primeiro relato de bloqueio de ramo esquerdo intermitente relacionado a esforços. O paciente apresentava palpitações e sensação de dor no precórdio durante as crises. Entretanto, a angiocoronariografia não foi realizada devido à tecnologia disponível naquela época.^1^ Em 1976, Vieweg et al.,^2^ relataram o primeiro caso de bloqueio de ramo esquerdo associado com angina de esforço, com evidências angiográficas de artérias coronárias normais. Embora tenha sido feito um diagnóstico de angina, foram observadas características atípicas: início e fim abruptos da dor, concomitantemente ao BRE e após o seu desaparecimento, respectivamente.^2^ Em 1982, Virtanen et al.,^3^ conduziram um estudo com 7 pacientes portadores de bloqueio de ramo esquerdo recente e dor precordial durante o teste de esforço, todos com angiocoronariografias normais. Nesse estudo, foi avaliado o padrão da dor apresentada pelos pacientes. Em todos os casos, a dor foi considerada atípica por conta do início e fim abruptos.^3^ Posteriormente, novos casos foram relatados, e a essa condição deu-se o nome de síndrome do bloqueio de ramo esquerdo doloroso.

Os mecanismos da síndrome do BRE doloroso são pouco claros. A possibilidade de isquemia de demanda resultante de lesões ou espasmos coronários foi inicialmente considerada uma possível causa para essa síndrome, mas logo essa suposição se mostrou incorreta. O início/fim imediatos da dor são incompatíveis com isquemia.^4^ A nitroglicerina se mostrou ineficaz^2^ e, às vezes, induziu o BRE devido à taquicardia. Muitas vezes, o resultado do exame de imagem nuclear era negativo e o vasoespasmo também havia sido descartado.^5 , 6^

A melhor teoria até agora é a proposta por Virtanen et al.,^3^ que, por meio da avaliação de ventriculografias, especulou que a dor poderia ser induzida pelo movimento sistólico anormal do septo. A presença do eixo inferior em uma série de casos de maneira uniforme fez com que os autores presumissem a existência de um padrão de contratilidade específico. Shvlikin et al.,^7^ propuseram critérios para o diagnóstico da síndrome do BRE doloroso ( Tabela 1 ).^7^

**Tabela 1:** – Critérios para a síndrome do BRE doloroso

Início abrupto de dor torácica com desenvolvimento do BRE
Resolução concomitante dos sintomas com resolução do BRE (ocasionalmente ausente)
ECG de 12 derivações normais antes e após o BRE
Ausência de isquemia miocárdica durante a prova de estresse funcional
Função ventricular esquerda normal e ausência de outras condições que possam explicar os sintomas
Relação S/T <1,8 em derivações precordiais e eixo inferior

*Critérios propostos para o diagnóstico da síndrome do BRE doloroso. Adaptado de Shvilkin.^7^*

De forma semelhante à onda T da memória cardíaca de pacientes com marcapasso, o BRE crônica apresenta ondas T de menor amplitude do que a BRE aguda. Em estudo prospectivo, uma relação S/T < 2,5 em derivações precordiais se mostrou eficaz (100% sensibilidade e 89% especificidade) para distinguir entre o BRE de início recente ou crônico,^8^ um dos itens dos critérios propostos na Tabela 1 .

A paciente a qual se refere este artigo foi encaminhada ao EEF por conta de uma hipótese equívoca de taquicardia supraventricular com aberrância. Durante o estudo, com a estimulação atrial contínua, tivemos a oportunidade de registrar o momento exato do bloqueio de ramo esquerdo e a imediata queixa sobre a mesma dor previamente referida pela paciente como sendo crônica.

**Figura 3:**
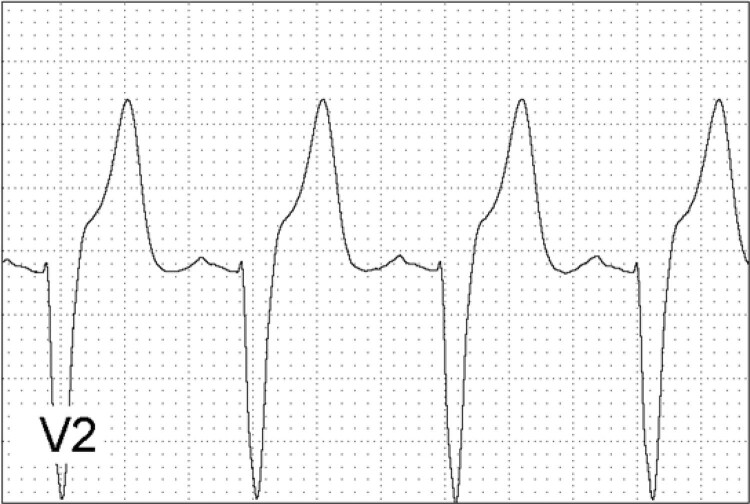
- Relação S/T < 1,8 em V2.

Ao traçarmos uma comparação com os critérios propostos por Schvilkin et al.,^8^ verificamos que nosso caso se encaixa em todos os critérios, com a exceção de um: o “critério do eixo inferior”. Entretanto, outras publicações também mostraram um complexo QRS superior.^9 , 10^ A relação S/T foi igual a 1,33 em V2 ( Figura 3 ), compatível com um BRE de início agudo. A paciente apresentou início abrupto de dor, conforme registrado pelos membros da nossa equipe no laboratório de eletrofisiologia. O desaparecimento dos sintomas ocorreu imediatamente após o desaparecimento do BRE. O ECG basal de 12 derivações estava normal. Um teste de esforço descartou a isquemia miocárdica e a angiotomografia revelou artérias coronárias normais. Tanto a ecocardiografia quanto a ressonância cardíaca estavam normais, com exceção das causas secundárias da angina.

## Conclusão

Relatamos um caso de BRE doloroso de uma paciente encaminhada ao EEF. O início abrupto da dor assim que o bloqueio do ramo esquerdo ocorre é incompatível com a isquemia. Além disso, a paciente foi submetida a exames que descartaram o comprometimento coronário e miocárdico. A melhor hipótese para a fisiopatologia dessa síndrome é a dissincronia dolorosa do coração resultante do BRE de início agudo. Até onde sabemos, este é o primeiro relato de caso sobre essa síndrome em uma revista médica brasileira.
