# Supplementary material for: Síndrome do Bloqueio de Ramo Esquerdo Doloroso em Paciente Encaminhada para Estudo Eletrofisiológico: Um Relato de Caso
Source: Arq Bras Cardiol. 2020 May 11;114(4 Suppl 1):34–7. [Article in Portuguese] doi: 10.36660/abc.20190295 (PMC8149111; doi:10.36660/abc.20190295)
Supplement: Translation

# Translation: Painful Left Bundle Branch Block Syndrome in a Patient Referred to Electrophysiologic Study: A Case Report

**Authors:** José Nunes de Alencar, Marcel Henrique Sakai, Saulo Rodrigo Ramalho de Moraes, Elano Sousa da Frota, Claudio Cirenza, Angelo Amato Vincenzo de Paola

**Affiliations:** 1 Brazil Universidade Federal de São Paulo Escola Paulista de Medicina, São Paulo, SP - Brazil

**Keywords:** Heart Block, Chest Pain, Coronary Artery Diseases/physiopathology, Cardiac Electrophysiology, Electrocardiography, Ecocardiography

## Introduction

The development of chest pain associated with intermittent left bundle branch block (LBBB) in the absence of coronary artery disease has been described in the literature as painful left bundle branch block syndrome. The mechanism responsible for the chest pain is unknown, but the main current hypothesis is related to acute cardiac dyssynchrony.

In this syndrome, the LBBB occurs when the cycle length is equal to or less than the refractory period of the left bundle, mainly during physical effort. The chest pain in the case of the painful LBBB syndrome may range from a mild discomfort to a disabling condition.

This report describes the case of a patient with typical rate-dependent LBBB associated with chest pain who was referred to electrophysiologic study (EPS) without evidence of arrhythmias.

## Case Report

A 41-year-old woman with a history of controlled hypertension and a 2-year history of palpitations associated with chest pain triggered by minimal efforts during daily activity, which lasted up to 2 hours. The chest pain was described as a pressure sensation that radiated to the left arm associated with nausea and dyspnea. The episodes were characterized by sudden onset, without any prodromes, with spontaneous improvement. She was initially treated with atenolol 25 mg bid, with partial relief of symptoms. There was no family history of unexplained syncope or sudden cardiac death. Her physical examination was unremarkable. The 12-lead electrocardiogram (ECG) during crisis revealed a wide complex tachycardia with complete left bundle branch block (LBBB), with inferior axis and a P wave compatible with sinus rhythm. Even so, the patient was referred to EPS, which did not evidence arrhythmogenic substrates.

However, at the start of a 600 miliseconds continuous atrial pacing, a rate-dependent LBBB was noted. Immediately succeeding the blockade of the LBBB, the patient, who was not maintained under sedation, started to complain about the same symptoms already described. The LBBB persisted during some minutes and relieved itself, concomitant to the relief of pain. The ECG is shown in Figure 1 . It is a typical third degree LBBB with a 138 ms QRS complex duration, superior axis and a sinus P wave.

**Figure 1:** - 12-lead ECG of the onset of pain immediately after left bundle branch block

The basal 12-lead ECG was normal ( Figure 2 ). The 24-hour ECG Holter monitoring revealed that basal HR was between 56 and 116 bpm during daily activities, with no evidence of LBBB. Transthoracic echocardiography and cardiac magnetic resonance both showed normal systolic function with no myocardial or valve pathologies. All cardiac chambers were normal in size. A stress test evidenced the development of left bundle branch block associated with thoracic pain. The CT angiography discarded coronary artery disease and myocardial perfusion defects on dypiridamole. Currently, the patient is receiving atenolol 50 mg bid and no recurrence of palpitations or chest pain was evidenced at the 6-month follow-up.

**Figure 2:** - Normal basal 12-lead ECG.

## Discussion

In 1946, the first report on intermittent left bundle branch block induced by effort was published. The patient presented with palpitations and aching feeling in precordium during crises. However, the coronary angiography was not performed due to the technology available at the time.^1^ In 1976, Vieweg et al.^2^ reported the first case of left bundle branch block associated with angina of effort, with angiographic evidence of normal coronary arteries. Although the patient was considered to have angina, atypical characteristics were present: sudden onset and offset, concomitantly with LBBB and after its disappearance, respectively.^2^ In 1982, Virtanen et al. carried out a study with 7 patients with new left bundle branch block and chest pain during exercise test, all of them with normal coronary angiography. In this study, the patient’s pain pattern was evaluated and considered as atypical pain, because of sudden onset and offset.^3^ Subsequently, new cases were reported, and this condition was called painful left bundle branch block syndrome.

The mechanisms of painful LBBB syndrome are unclear. The possibility of demand ischemia due to coronary lesions or spasms was initially considered as a possible cause for this syndrome. However, this assumption was soon proved wrong. Immediate onset/offset of pain is incompatible with ischemia.^4^ Nitroglycerin was proved to be ineffective^2^ and sometimes induced LBBB due to tachycardia. Nuclear imaging is frequently negative in these patients and vasospasm has also been discarded.^5 , 6^

The best theory so far was proposed by Virtanen et al.,^3^ who speculated that the pain could be induced by abnormal systolic motion of the septum in ventriculography. An inferior axis which presented uniformly in a series of cases made the authors hypothesize that this could reflect a specific contractility pattern. Shvlikin et al.^7^ proposed criteria to the diagnosis of painful LBBB syndrome ( Table 1 ).^7^

**Table 1:** – Painful LBBB syndrome criteria

Abrupt onset of chest pain with development of LBBB
Simultaneous resolution of symptoms with resolution of LBBB (occasionally absent)
Normal 12-lead ECG before and after LBBB
Absence of myocardial ischemia during functional stress test
Normal left ventricular function and absence of other conditions that may explain the symptoms
Low precordial S/T wave ratio (< 1.8) and inferior axis

*Criteria proposed for the diagnosis of the painful LBBB syndrome. Adapted from Shvilkin.^7^*

Similar to the memory T waves of paced patients, chronic LBBB has lower amplitude T waves than acute LBBB. In a prospective study, a S/T ratio < 2.5 in precordial leads proved to be useful (100% sensitivity and 89% specificity) to help distinguish between new-onset or chronic LBBB,^8^ which refers to one of the items of the criteria proposed in Table 1 .

The patient described in this paper was referred to EPS because of the mistaken hypothesis of supraventricular tachycardia with aberrancy. During the study, with continuous atrial pacing, we had the opportunity to register the exact moment of left bundle branch blockade and the immediate complain about the same pain she referred to as chronic.

Regarding the criteria proposed by Schvilkin et al.,^8^ our case matches all but one criterion: the “inferior axis criterion”. However, other publications evidenced superior QRS complex axis as well.^9 , 10^ The S/T wave ratio was 1,33 in V2 ( Figure 3 ), which is compatible with acute onset LBBB. The patient had abrupt onset of pain, as registered by the members of our team in our electrophysiology lab; the resolution of the symptoms occurred immediately after the resolution of the LBBB; The basal 12-lead ECG was normal. A stress test discarded myocardial ischemia and the CT angiography evidenced normal coronary arteries. Echocardiography and cardiac resonance were both normal, excluding secondary causes of angina.

**Figure 3:** - S/T ratio < 1.8 in V2.

## Conclusion

We reported the case of a patient with painful LBBB who was referred to electrophysiology study. The abrupt onset of pain immediate after left bundle blockade is incompatible with ischemia and the patient underwent exams that discarded coronary and myocardial involvement. The best hypothesis for the pathophysiology of this syndrome is painful desynchrony of the heart due to acute onset LBBB. To our knowledge, this is the first case report of this syndrome in a Brazilian medical journal.
